# Women’s status and experiences of mistreatment during childbirth in Uttar Pradesh: a mixed methods study using cultural health capital theory

**DOI:** 10.1186/s12884-016-1124-4

**Published:** 2016-10-28

**Authors:** May Sudhinaraset, Emily Treleaven, Jason Melo, Kanksha Singh, Nadia Diamond-Smith

**Affiliations:** 1 Department of Epidemiology and Biostatistics and Global Health Sciences, University of California, San Francisco. 550 16th Street, Box 1224, San Francisco, CA 94158 USA; 2 Department of Social and Behavioral Sciences, University of California, San Francisco. 3333 California St., San Francisco, CA 94143 USA; 3 Division of Global Public Health, Department of Medicine, University of California, San Diego. 9500 Gilman Drive, La Jolla, CA 92093 USA; 4 Foundation for Research in Health Systems, 214, Sydicate House, Inderlok, Delhi, India

**Keywords:** Mistreatment, Disrespectful care, India, Quality of care, Women, Slum, Cultural health capital

## Abstract

**Background:**

Mistreatment of women in healthcare settings during childbirth has been gaining attention globally. Mistreatment during childbirth directly and indirectly affects health outcomes, patient satisfaction, and the likelihood of delivering in a facility currently or in the future. It is important that we study patients’ reports of mistreatment and abuse to develop a deeper understanding of how it is perpetrated, its consequences, and to identify potential points of intervention. Patients’ perception of the quality of care is dependent, not only on the content of care, but importantly, on women’s expectations of care.

**Methods:**

This study uses rich, mixed-methods data to explore women’s characteristics and experiences of mistreatment during childbirth among slum-resident women in Uttar Pradesh, India. To understand the ways in which women’s social and cultural factors influence their expectations of care and consequently their perceptions of respectful care, we adopt a Cultural Health Capital (CHC) framework. The quantitative sample includes 392 women, and the qualitative sample includes 26 women.

**Results:**

Quantitative results suggest high levels of mistreatment (over 57 % of women reported any form of mistreatment). Qualitative findings suggest that lack of cultural health capital disadvantages patients in their patient-provider relationships, and that women use resources to improve care they receive. Participants articulated how providers set expectations and norms regarding behaviors in facilities; patients with lower social standing may not always understand standard practices and are likely to suffer poor health outcomes as a result. Of importance, however, patients also blame themselves for their own lack of knowledge.

**Conclusions:**

Lack of cultural health capital disadvantages women during delivery care in India. Providers set expectations and norms around behaviors during delivery, while women are often misinformed and may have low expectations of care.

## Background

Maternal mortality remains a significant public health issue globally, and a growing literature suggests that mistreatment of women in healthcare settings must be addressed in order to improve mortality outcomes [1–3]. Mistreatment is a broad term that encompasses women’s experiences with disrespect, physical and verbal abuse, neglect, as well as deficiencies in the facility environment and broader health system [4]. Mistreatment directly and indirectly effects health outcomes, patient satisfaction, and the likelihood of delivering in a facility currently or in the future [5, 6]. Past experiences with mistreatment may deter women from attending a facility even in instances where they experience complications [3]. Sub-standard quality of care contributes towards maternal mortality; for example, a study assessing all maternal deaths in tertiary care hospitals in Sri Lanka found that the majority of maternal deaths were preventable, with 79 % attributable to substandard care [7]. Beyond poor health outcomes, a study in Tanzania suggests that women who experience disrespectful or abusive care reported less satisfaction with their delivery, perceived quality of care for delivery, and were less likely to deliver in the same facility [8]. Analyses suggest that were respectful care and availability of drugs/equipment to improve, that the proportion of women preferring a facility delivery would increase from 43 to 88 % [5].

Mistreatment of women in healthcare settings during childbirth has been gaining attention globally [9]. In India, a number of studies report women’s experiences of mistreatment in reproductive and maternal health services, including delivery. Indian women report being left unsupported, shouted at, and slapped during facility deliveries; women report that they were not given information about what treatment they were receiving and why they were receiving it [10]. At the facility level, delivery environments may be chaotic and unsafe in India [11].

Paradoxically, past studies have found that women with higher education and socioeconomic status report higher levels of mistreatment and lower perceived quality of care [12]. At a national level in India, states with the highest level of literacy and longevity tend to also report the highest rates of morbidity [13], highlighting the difference between observed versus perceived health. In healthcare facility settings, while women who are better off tend to *report* lower levels of quality care, in direct observation studies, higher socioeconomic status women are in fact *observed* to receive higher quality of care [14]. Past research in India has found that literate women were more likely to have their procedures explained to them [10]. Patients’ perception of the quality of care is dependent on the content of care, but importantly, also on women’s own expectations of care. In a systematic review of the patient satisfaction, Sitzia and Wood [15] identified patient expectations as a major determinant of satisfaction. Patients’ expectations are often shaped by their socio-demographic characteristics [15].

Examining women’s lived experiences with maternal health services is critical to identifying drivers of access and utilization of care and to improve quality of care [10]. While the qualitative literature has increasingly reported experiences of mistreatment globally, there is limited quantitative research measuring the magnitude of mistreatment being experienced and associated risk factors. To date, the qualitative literature on mistreatment has under-theorized determinants and consequences of mistreatment.

### Linking women’s expectations to reports of mistreatment: cultural health capital theory

To understand the ways in which women’s social and cultural characteristics influence their expectations of care and consequently their perceptions of respectful care, we adopt a Cultural Health Capital (CHC) framework. CHC is defined as a “specialized set of cultural skills, behaviors and interactional styles that are valued and leveraged as assets by both patients and providers in clinical encounters” [16]. Patient strategies include knowledge of medication and health conditions, an ability to communicate that knowledge efficiently, the ability to adjust one’s interactional style, and utilizing organizational skills in healthcare settings (i.e. writing notes on which drugs to take and when to take them). These cultural skills and resources are critical to the ability of patients and providers to effectively engage and communicate with one another. Patients with greater CHC more easily engage with providers and navigate health systems, potentially deriving greater benefit from care [17].

Rooted in Bourdieu’s concepts of cultural capital [18, 19], this framework suggests that CHC develops through repeated experiences of health-related practices. First, rather than engaging in standardized, purposeful interactions, many women are likely to respond in “habitual ways that are rooted in their experiences, schemes of thought and perception, long-lasting ways of organizing action, and their general sensibilities about how the world works” [16]. In other words, the ways in which women respond to their providers, and even their expectations of providers, are deeply rooted in past social experiences with formal institutions, including healthcare systems. In India, many women deliver outside formal health facilities, and in Uttar Pradesh, only 27 % had three or more antenatal care visits [20]. If women have never been to a healthcare facility prior to their delivery, as is common in India, their expectations is likely to differ significantly from a woman who has had past experiences with formal healthcare, such as attending antenatal care. Therefore, cultural health capital is not an innate quality; rather, it is a learned set of skills based on practice. Similar to social and cultural capital, CHC has been conceptualized as a resource. Similar to Bourdieu’s conceptualization of cultural capital, each experience in a healthcare setting becomes an opportunity to gain additional cultural health capital, including skills, social competencies, and decision-making techniques [16]. However, some individuals lack the agency to develop and deploy these strategies in health interactions, oftentimes related to larger social structures and inequalities, such as lower socioeconomic status, lower literacy, and lower social standing in relation to gender, sex, age, or in India, class designations.

CHC is relational and driven by institutional power dynamics. While patient-provider interactions can be thought of as ‘give-and-take’, or jointly determined, in poor contexts, providers are much more likely to play a significant role in how CHC is deployed and cultivated [21]. Rather than only respond to CHC that patients deploy, providers may also encourage patients in healthcare settings through signals and communication styles [16]. Conversely, providers may also discourage patient CHC, and in the US context, clinical encounters have been shown to disadvantage minority patients [22]. In developing contexts, providers may abuse patients to assert control as a means of distancing themselves from patients and maintain ideas of self-identity and power [23].

Because of past poor experiences, or a lack of experience with formal healthcare altogether, poorer and lower-standing women are less likely to deploy CHC. Based on this framework, we hypothesize that women of lower social standing have inculcated lower levels of expectations in regards to health; consequently, they are less likely to report mistreatment, regardless of whether it occurred or not. Understanding CHC in the context of urban, developing country settings is increasingly important given overburdened health systems, large urban populations, and the significant cultural heterogeneity of women in facilities. To our knowledge, CHC has only been conceptualized in the US context [16, 17, 21]. This study expands the concept of cultural health capital to the Indian context, among slum-resident women who deliver in facilities. Furthermore, it expands CHC to women’s delivery care, an experience deeply rooted in traditional and cultural practices.

This study explores factors associated with Indian women’s report of mistreatment from both a qualitative and quantitative perspective, and provides a theoretical exploration of how mistreatment is perpetrated, its drivers, and its consequences. Understanding mistreatment through mixed-methods allows women to report not only what types of disrespect are occurring, but also aids in understanding how and why they are experiencing it. We utilize mixed-methodology data to explore socio-demographic predictors of mistreatment in facility delivery, and how cultural health capital operates in this context with respect to mistreatment. The data comes from a sample of low-income and low-status women living in slums in Lucknow. Specifically from qualitative data, we are interested in women’s description of forms of cultural health capital and how these relate to care, as well as the strategies that women may use to improve delivery care.

## Methods

The study sites for all data collection activities, including surveys and focus group discussions, were slums of Lucknow city of Uttar Pradesh, India. A list of slums was obtained from the District Urban Development Authority (DUDA). Thirty-eight slums were selected randomly as this was estimated to be large enough to recruit the desired sample size based on prior power calculations of the likely number of women who would match our eligibility criteria within the population. There are a total of 713 slums in Lucknow and 40.2 % of the population of Lucknow lives in slums [24]. A number of past studies have found that maternal and reproductive health outcomes were worse for women living in slum areas of Lucknow compared to other parts of Lucknow, for example breastfeeding [25]. All surveys and discussions were completed in Hindi and occurred in April and May of 2015.

### Quantitative sample

A total of 760 respondents were enrolled in the quantitative study. To be eligible, women had to be between 18 and 30 years of age, have at least one child currently under the age of five, and live in the slum area. To select households, the research team first made a listing of all households in the study area and then randomly selected households to visit to see if they had an eligible woman (through a series of questions about eligibility). Households were approached until the desired sample size of 380 migrants and 380 non-migrants was reached.

The quantitative analysis is restricted to 392 women who reported that their most recent birth took place in a health facility, in order to ensure reports of mistreatment were first hand. Women who had not delivered in a facility were asked to report if they heard second-hand about experiences of mistreatment, but these women were excluded from this analysis.

Surveys took about one hour to complete. The survey included a series of demographic questions as well as a birth history. Detailed data about the most recent birth, including measures of mistreatment, support, experiences and future preferences were collected. These measures have been previously developed and used in other settings [26, 27].

### Qualitative sample

Focus group participants were recruited through Anganwadi centers with the help of community health workers. Study researchers randomly selected four slums from the list of 38 slums for the larger quantitative survey to conduct qualitative focus group discussions. Study researchers first approached community health workers in each slum, typically trusted leaders in the community, and described the study, including the objectives and risks. Researchers then asked for permission to hold a focus group discussion as well as support in recruiting women. Inclusion criteria for participants included being between the ages 18 to 30, had at least one child currently under the age of five, lived in the slum area, and self-identified as a migrant (no set definition, based on a self-identification).

A total of eight focus group discussions were conducted, including four groups with female participants and four groups with male participants. The present analysis is restricted to women in order to limit to first-hand accounts of experiences around facility deliveries, specifically using data from the four female focus groups. In total, the qualitative sample includes 26 women. Focus groups were led by a trained, same-sex moderator and supported by a same-sex note-taker. All discussions were tape-recorded. Approximately 6-8 participants were included in each group discussion. Focus groups utilized a semi-structured interview guide, which was pre-tested through a pilot group discussion. The interview guide included questions about migration, social connections with villages of origin and in Lucknow, experiences with mistreatment during delivery, health knowledge, access to health services, and gender norms and beliefs. Focus groups were held at a community center in a private room in each slum, and lasted about one and a half to two hours each. Each focus group was led by a trained moderator and assisted by a note taker. At the conclusion of each focus group, participants completed a short questionnaire that included information about socio-demographic characteristics, their household, and information about migration.

### Analyses

#### Quantitative analysis

We present descriptive and bivariate statistics. The outcome of interest is based on a summative measure of perceived mistreatment. The questionnaire asked women about eleven distinct types of mistreatment including: discrimination based on race, ethnicity, or ability to pay; physical abuse (slapping or hitting); verbal abuse (insult and shouting); threatening to withhold treatment; lack of information about care provided; ignoring or abandoning patient when in need; delivering alone; denying choice of position during delivery; birth companion(s) not allowed; request or suggestion for informal payments or bribes for better care; or unnecessary separation from baby after birth. Potential covariates explored included age (16-19 years; 20-24 years; 25-30 years); migration status (non-migrant; urban to urban; rural to urban); caste (other; scheduled caste; scheduled tribe; other backward caste),^1^ wealth quartile; and education level (illiterate/no education; primary; secondary or more). Wealth quartiles were determined based on principle components analysis using a listing of household durable goods [28].

#### Qualitative analysis

Focus group discussions were transcribed into Hindi and translated to English. Focus group discussions were analyzed using an open coding approach in Atlas.ti. Several authors reviewed the focus group transcripts to identify themes related to mistreatment prior to coding. A code list was created iteratively during the coding process, applied to new interviews, and refined throughout, as were code definitions and hierarchies [29]. The code list, definitions, and hierarchies were cross-checked with the transcripts by three researchers to ensure validity. This analysis utilizes codes related to experiences of delivery, accessing healthcare and health knowledge. The analysis is supplemented by codes relating to migration and life in villages of origin.

### Ethical review, consent, and permissions

Eligible woman were read an informed consent, and if interested, gave verbal consent to participate. All study documents and procedures were reviewed and approved by the Institutional Review Boards at the University of California, San Francisco (UCSF) and Foundation for Research in Health Systems (FRHS) in India.

## Results

We first report demographic characteristics of both the quantitative and qualitative data. Next, we present bivariate analyses of women’s report of mistreatment across different social and demographic characteristics. We then present qualitative data that describes how women experience mistreatment in the Indian context, cultural health capital in delivery care, and the resources that patients may employ to improve quality of care.

### Demographic characteristics: quantitative surveys

A total of 759 women completed the quantitative survey, including 392 women who reported delivering at a health facility for their most recent birth. Table 1 shows the socio-demographic characteristics of the sample of women delivering at a health facility. Women who delivered at a health facility have a mean age of 25.3 years (*SD* = 3.1 years), with the greatest proportion of women ages 25 to 30; less than five percent of the sample is 18 to 20 years of age. Over two-thirds of the sample is Hindu. A majority of women are members of a scheduled class or otherwise backward class, and 11.7 % are members of a scheduled tribe. Over 99 % of the sample is currently married, with a mean age at marriage of 17.2 years (*SD* = 2.4 years). Almost three-quarters of women in the sample were married between ages 13 and 18. Many women have little to no education; 36.5 % are illiterate or have no formal education, while 37.8 % have some or complete primary education. However, 25.8 % of women attended at least some secondary or post-secondary education. Women who delivered at a health facility tended to be slightly wealthier than the full sample of women; 32.0 % are in the richest wealth quartile. This sample includes 22.5 % of women in the lower middle and 25.8 % of women in the upper middle quartiles, and 19.7 % of women in the poorest quartile. Women in this restricted sample live in households with a mean of 4.8 members (*SD* = 1.7 members).

**Table 1:** Selected socio-demographic sample characteristics of participants

	Quantitative survey	Focus group discussions
	*N* = 392	*N* = 26
Age (mean +/- SD)	25.3 years (3.1)	24.9 years (3.1)
Less than 20 years	19 (4.9 %)	1 (3.9 %)
20 – 24 years	134 (34.2 %)	9 (34.6 %)
25 – 30 years	239 (60.9 %)	16 (61.5 %)
Religion		
Hindu	269 (68.62 %)	13 (50.0 %)
Muslim	123 (31.8 %)	13 (50.0 %)
Class		
Scheduled class	131 (33.4 %)	2 (7.7 %)
Schedule tribe	46 (11.7 %)	2 (7.7 %)
Other backwards caste	159 (40.6 %)	22 (84.6 %)
Other	56 (14.3 %)	0 (0.0 %)
Currently married	390 (99.5 %)	26 (100.0 %)
Age at marriage (mean +/- SD)	17.2 years (2.4)	15.5 years (4.0)
Less than 13 years	11 (3.1 %)	7 (26.9 %)
13 – 18 years	289 (73.7 %)	15 (57.7 %)
19 – 25 years	91 (23.2 %)	3 (11.5 %)
26 – 30 years	0 (0 %)	1 (3.9 %)
Number of household members (mean +/- SD)	4.8 members (1.7)	5.4 members (3.2)
Number of births (mean +/- SD)	2.1 births (1.1)	2.7 births (1.3)
Educational attainment		
None/illiterate	143 (36.5 %)	14 (53.9 %)
Primary	148 (37.8 %)	7 (26.9 %)
Secondary or higher	101 (25.8 %)	5 (19.2 %)
Wealth quartile		
Poor	77 (19.7 %)	-
Lower middle	88 (22.5 %)	-
Upper middle	101 (25.8 %)	-
Rich	125 (32.0 %)	-
Site of delivery for most recent birth		
Government hospital	82 (20.9 %)	-
Primary health center	196 (50.0 %)	-
Private hospital	82 (20.9 %)	-
Private clinic	32 (8.2 %)	-
Accompanied to delivery		
Husband	344 (87.8 %)	-
Mother-in-law	177 (45.2 %)	-
Mother	191 (48.7 %)	-
Sister	116 (29.6 %)	-
Friend	137 (34.9 %)	-
Other	92 (23.5 %)	-

Women who delivered in a health facility report a mean of 2.1 births in their lifetime (*SD* = 1.1 births). Public sector primary health centers are the most common place of delivery, where 50.0 % of the sample delivered. Women also report delivering at government hospitals and private hospitals; 20.9 % of the sample delivered at each. Finally, 8.2 % of women report delivering at a private clinic. A majority of women, 87.8 %, were accompanied by their husband to their most recent delivery. Nearly half of women were accompanied by their mother (48.7 %) or mother-in-law (45.2 %). Women report that they were also accompanied by their sister (29.6 %), a friend (34.9 %), or another person (23.5 %).

### Demographic characteristics: qualitative sample

Socio-demographic characteristics of 26 female focus group discussion participants are reported in Table 1. The mean age of focus group participants, 24.9 years (*SD* = 31 years) is slightly younger than the mean age of quantitative survey respondents, though the distribution of ages is similar in the two samples. Half of focus group participants are Hindi, and half are Muslim. A high proportion of focus group participants, 84.6 %, are members of an otherwise backwards class. The remainder is members of a scheduled class (7.7 %) or scheduled tribe (7.7 %). All participants are currently married, and were on average 15.5 years of age when they married (*SD* = 4.0 years). Focus group participants tended to marry at younger ages compared to quantitative survey respondents. Respondents live in households with an average 5.4 members (*SD* = 3.2 members), and have had an average of 2.7 births in their lifetime (*SD* = 1.3). Over half of focus group participants have no formal education (53.9 %). Just over one-quarter has attended at least some primary school (26.9 %), while 19.2 % have attended some secondary or post-secondary school.

### Quantitative findings

#### Prevalence of mistreatment

Over half of survey respondents (54.7 %) report experiencing any type of mistreatment during a facility delivery. Among the eleven forms of mistreatment shown in Table 2, verbal abuse is the most commonly reported behavior (28.6 %), followed by a request for payments or bribes (24.2 %). Not allowing a companion (19.6 %), discrimination (16.8 %), and physical abuse (15.5 %) are the next most prevalent forms of disrespect or abuse reported. About 10 % of the sample reports experiencing threats to withhold treatment, being abandoned or ignored, delivering alone, or being denied their preferred choice of position for delivery. A lack of information (4.6 %) and unnecessary separation from the baby (4.3 %) are the least prevalent behaviors reported.

**Table 2:** Reported mistreatment among quantitative sample (*N* = 392)

Type of mistreatment	Yes	No	Don’t know
Discrimination	66 (16.8 %)	282 (71.9 %)	44 (11.2 %)
Physical abuse	61 (15.5 %)	284 (72.5 %)	47 (12.0 %)
Verbal abuse	112 (28.6 %)	240 (61.2 %)	40 (10.2 %)
Threats to withhold treatment	48 (12.2 %)	294 (75.0 %)	50 (12.8 %)
Lack of information	18 (4.6 %)	311 (79.6 %)	63 (16.1 %)
Abandoned or ignored	40 (10.2 %)	298 (76.0 %)	54 (13.8 %)
Delivered alone	41 (10.5 %)	296 (75.5 %)	55 (14.0 %)
Choice of position denied	41 (10.5 %)	286 (72.9 %)	65 (16.6 %)
Companion not allowed	77 (19.6 %)	268 (68.4 %)	47 (12.0 %)
Requested payment or bribe	95 (24.2 %)	251 (64.0 %)	46 (11.7 %)
Unnecessary separation from baby	17 (4.3 %)	311 (79.3 %)	64 (16.3 %)

#### Bivariate associations of disrespectful behaviors with socio-demographic characteristics

Older women, compared to younger, were significantly more likely to report being asked to pay bribes (Table 3). Age was not associated with any of the other outcomes. Non-migrants were significantly more likely to report not being allowed a companion of their choice and had a higher mean disrespect score compared to women who migrated. Richer women, compared to poorer women, were significantly more likely to report verbal abuse, not being allowed a companion of their choice and had a higher mean disrespect score. Lower caste women were more likely to report many types of mistreatment compared to women of other caste, including: discrimination, verbal and physical abuse, being abandoned and ignored, requested to pay bribes, and the overall disrespect score. There were no significant differences in mistreatment by education status.

**Table 3:** Bivariate analysis of significance by demographic factors, percent reporting having experienced each form of mistreatment (Chi square, * = *p* < =0.05)

		Discrimination (%)	Physical abuse (%)	Verbal abuse (%)	Threats to withhold treatment (%)	Lack of information (%)	Abandoned or ignored (%)	Delivered alone (%)	Choice of position denied (%)	Companion not allowed (%)	Requested payment or bribe(%)	Unnecessary separation from baby (%)	Total Mistreatment Score (mean)
Total N reporting mistreatment													
		132	121	214	89	35	65	85	76	140	167	34	Range 1-10
Age	16-19	6.8	5.8	4.7	5.6	8.6	3.1	5.9	6.6	5.7	4.6	2.9	1.58
	20-24	39.4	33.1	36.9	34.8	31.4	33.8	31.8	38.2	30	39.4	20.6	2.31
	25-30	53.8	61.2	58.4	59.6	60	63.1	62.4	55.3	64.3	56	76.5	1.90
	Chi sq										*		
Migrant	Non	55.3	55.4	50.5	49.4	51.4	56.9	57.6	57.9	65	50.9	64.7	2.40
	Rural to Urban	31.8	33.1	37.9	37.1	40	27.7	32.9	31.6	29.3	36.6	26.5	1.72
	Urban to Urban	12.9	11.6	11.7	13.5	8.6	15.4	9.4	10.5	5.7	12.6	8.8	1.63
	Chi sq									*			*
Wealth (quartile)	1	25	24	20.1	27	31.4	12.3	23.5	27.6	20.7	16.6	26.5	1.06
	2	27.3	26.4	24.8	27	22.9	27.7	32.9	30.3	30.7	26.3	17.6	2.06
	3	21.2	21.5	23.8	19.1	22.9	29.2	21.2	19.7	22.9	24.6	41.2	1.90
	4	26.5	28.1	31.3	27	22.9	30.8	22.4	22.4	25.7	32.6	14.7	2.72
	Chi sq			*						*	*		*
Caste	Other	6.1	6.6	7	4.5	2.9	6.2	10.6	10.5	9.3	4.6	0	1.54
	SC	22	22.3	22.4	34.8	45.7	20	36.5	31.6	25.7	16.6	11.8	1.27
	ST	18.2	16.5	11.7	13.5	14.3	18.5	1.2	2.6	12.9	21.1	23.5	2.97
	OBC	53.8	54.5	58.9	47.2	37.1	55.4	51.8	55.3	52.1	57.7	64.7	2.55
	Chi sq	*	*	*			*				*		*
Education	None	52.3	55.4	50	56.2	60	43.1	50.6	56.6	54.3	51.4	61.8	2.10
	Primary	33.3	29.8	35.5	27	28.6	36.9	34.1	30.3	30.7	28.6	26.5	1.80
	Secondary	14.4	14.9	14.5	16.9	11.4	20	15.3	13.2	15	20	11.8	2.26
	Chi sq												

## Qualitative findings

### Minimal and poor experiences with healthcare

Most migrant women had not had experiences with formal care in their villages of origin; many attributed this to social norms around care, or a lack of formal healthcare facilities in their villages of origin before they migrated. In addition, prior poor experiences are a deterrent to use, as many women actively considered the experiences of their friends and neighbors, as well as their own, in making decisions about whether and where to seek healthcare. Many women discussed a low quality of care in their villages of origin, which coupled with social and familial norms around care seeking, led to a lack of access to formal care.

“*Health facilities in villages are not good. Services are also not good like doctors won’t listen to patients’ problem properly or either they won’t give proper medicines*…*some family member does not allow consulting doctors or they would try to suggest self-medication. They would underestimate health problems leading to no medication or consulting doctor.”* (Age 20, from rural area of another state)

Many women were unsure where they might seek care in Lucknow. For example, many illiterate women feel disadvantaged because they cannot understand even menial processes in healthcare. One respondent reports, “*I don’t know what nurses keep writing on that piece of paper. We don’t understand it*” (Age 29, from a rural area of another state). Among many respondents, a lack of information about health facilities and available services was cited as a primary reason for not seeking services.

“*We are for each other. We don’t usually go to hospitals as we don’t have any information related to maternal and child health*.” (Age 29, from a large city in Uttar Pradesh)

Multiple women agreed that not understanding whom to call in instances such as at the onset of labor, or more generally, how and when to utilize reproductive and maternal health care, deterred them from using facilities.

*“If we call an ambulance, government service, back home, it comes. Here, we don’t know whom to call for emergencies. We have to get a paid transport.”* (Age 25, from rural Uttar Pradesh)

*“We don’t have any one to inform us on health issues like some ASHA [community health worker] or ANM [trained nurse midwife]. They come here in our community only when polio rounds are going on.”* (Age 28, from rural Uttar Pradesh)

This lack of institutional support impedes access to available care for both preventive and curative care, and importantly, at delivery. A lack of education also presents a barrier to care for many respondents. Particularly among women who are illiterate, difficulties with reading instructions or directions to the hospital are a deterrent to attending a facility.

### Various forms of mistreatment occur at health facilities

Respondents commonly reported that the poor face discrimination and abuse in health facilities, which is a deterrent to their use. Specifically, a number of respondents felt the quality of care at government facilities was sub-standard for the poor compared to those of middle or higher socio-economic status, who received better treatment and faced fewer barriers to accessing care in the public sector. Among a majority of respondents, poverty is perceived as the most important cause in predicting who will experience disrespect or abuse. While some respondents felt migrants face discrimination, many reported that migrants received similar treatment as non-migrants, and refuted that migrants received poorer quality of care.

The types of mistreatment reported in focus group discussions are similar to those found in the quantitative survey. The most commonly reported type of abuse among focus group participants is verbal abuse, which includes “scolding” patients and shouting at them.

*“For [higher socio-economic status] government health facilities are very good. For us, poor people, it is not so good. We are abused. If you ask anything to them they would shout back at you. They think they are big shots and know each and everything. What would a poor man do in such situations? We have to keep mum.”* (Age 28, from rural Uttar Pradesh)

Here, she attributes verbal abuse to her lower status, and feels there is no recourse for this abuse. Beyond verbal abuse, several patients also described instances of physical abuse. Several respondents described paying bribes to receive proper treatment and access medications; if they are unable to pay such bribes, they do not receive treatment. One respondent describes an especially egregious bribe:

*“When my sister-in-law delivered, staff there started asking for money. They were asking for money because my sister-in-law had delivered a baby boy. They exchanged our baby boy with a girl. We had to pay each more than Rs.500 to get baby back and get discharged.”* (Age 25, from rural Uttar Pradesh)

While this represents an extreme example, bribes were characterized as commonplace in the course of care at public facilities. Finally, a majority of respondents perceived that the poor are delayed in receiving care at these facilities, and are forced to wait longer than others for treatment. Several respondents remarked that those of higher socio-economic status were able to avoid queues and receive more timely and higher quality care.

### Lack of cultural health capital influenced providers’ response

Many respondents recognized that health facilities have a specific set of social rules. However, marginalized respondents reported that while they recognize situations of mistreatment, they face difficulties in their ability to follow, or even identify or understand, these rules. Several respondents blamed themselves for their lack of cultural health capital:

*“This is our fault and not doctor’s…We don’t know many things. If we are literate and know to read, we would be able to identify different directions given at hospitals. So, it is vain to go there.”* (Age 29, from a small city in Uttar Pradesh)

Here, the respondent identifies a structural barrier to respectful, appropriate care: a lack of education. That is, in this setting, education or literacy, is an important instrumental resource that can improve the quality of care when leveraged in healthcare settings.

When respondents are unable to act in accordance with or unaware of these social rules, they may experience mistreatment by providers. One respondent describes such an instance, which upset the provider and resulted in verbal abuse.

*“Sometimes, when patients don’t listen and try to violate privacy of other patients, doctors get angry. Doctors request them to come one by one but clients don’t follow doctor. That time doctors have all rights to get angry. It is mistake of other patients that they are trying to breech confidentiality of the one who is in doctor’s cabin.”* (Age 25, from a rural area in another state)

The doctors have set expectations about how patients will or should act, and patients must utilize cultural health capital in these settings. Where they do not, they are at risk of mistreatment.

### Patients receive better treatment by leveraging resources

Respondents recognize that they can improve the quality of care received, and avoid mistreatment by utilizing specific resources, and using interactional styles that meet provider expectations. Patients who actively engage with providers are perceived to receive better and faster service. One respondent describes questioning providers as an interactional style that exemplified higher cultural capital.

*“If any patient comes later than us but keeps questioning hospital staff about each and everything, they are served first. As we don’t ask any questions to anyone (because we don’t know anything), we keep waiting.”* (Age 27, from rural Uttar Pradesh)

A strategy for accessing cultural health capital is to attend care with someone who is familiar with the providers and processes at a healthcare facility. By leveraging another person’s cultural health capital, respondents are able to receive care in a timelier manner, and are less subject to disrespect or abuse.

*“Yeah. If we have someone who knows to doctor at a government facility, we are provided with services a little faster than usual. But if we go alone, we face problems.”* (Age 28, from rural Uttar Pradesh)

Some respondents identified specific members of their family or community who were familiar with facilities, and asked that person accompany them when accessing services. Having someone accompany them to facilities increased their comfort in utilizing facility services. For example, one participant describes how her mother helped navigate the complicated system of receiving money for her delivery as well as her satisfaction with care.

*“I have no complaints. I was treated well. I went with my mother-in-law. Staff there know my mother-in-law.”* (Age 28, from rural Uttar Pradesh)

Several other women agreed that attending a facility with someone who is familiar with the ways of care improves the quality of their care. The use of patient advocates is an important mechanism through which marginalized women improved their cultural health capital. These advocates are described as community leaders who are familiar with the health system, suggesting they have greater cultural health capital that can be leveraged by those less experienced with formal care.

## Discussion

This mixed-methods study explores the social and cultural underpinnings of mistreatment in India using the theoretical lens of cultural health capital. In line with other studies, quantitative results suggest high levels of mistreatment, with over 57 % of women reporting any form of mistreatment. In line with other research, we found that wealthier women reported higher levels of disrespect. Non-migrant women also reported less disrespect compared to migrant women. This is commensurate with other studies that find that richer women were more likely to report abuse [12]. Past research in the developed world has found that migrants from developing countries face discrimination and disrespect in childbirth [30, 31]. This is the first study to our knowledge to explore this issue and find this relationship among internal migrants in a developing country.

However, we also found that lower caste women reported more mistreatment, which reflects past literature on the importance of caste in the Indian context. For example, prior studies found that scheduled tribe populations have higher mortality compared to other groups in India, even after controlling for other factors such as wealth [32]. Scheduled tribes are isolated and socially distanced from mainstream Indian (Hindu) society, often speaking different languages, living in more remote regions, and having different cultural practices [33]. It is therefore not surprising that this population perceives having experienced more outright discrimination than the other castes in this study. Indigenous populations in multiple countries report dissatisfaction with delivery services as a barrier to their use, as services did not allow for culturally preferred practices around childbirth [26]. In Peru, facility delivery among indigenous women increased after a facility-based intervention to better incorporate culturally preferred practices around childbirth [34]. It is additionally likely that providers and facility staff see these women as being less empowered or see scheduled tribe or otherwise backwards class (OBC) groups as more different from themselves, and therefore feel that they can demand bribes from these individuals.

There are possible explanations for the different findings for wealth vs. caste. It is critical to consider the composition of our sample, which was recruited from disadvantaged slum areas. Thus, the entire sample comprised of poor individuals, and the wealthy in our wealth quartile are fairly poor compared to the population of Lucknow. A more economically diverse sample representative of the greater population would have been able to assess whether, in fact, the truly wealthy, rather than the relatively wealthy, perceive less mistreatment. Another explanation may be that the mechanisms of disrespect on wealth and caste may differ. Cultural health capital theory suggests that wealthier women are more likely to have had repeated experiences with healthcare settings in the past [17]. Therefore, wealthier women have higher levels of expectations of care. Low caste, on the other hand, may be too difficult to overcome, given the historical and persistent cycle of disadvantage and discrimination that this group faces [33]. Future studies should disentangle potential mechanisms that may be at play regarding wealth and caste in India.

Our qualitative data further sheds light on women’s experiences of mistreatment. Of interest is that women are able to describe and recognize the demeaning care that the poor in particular receive. Cultural health capital theory suggests that there is a difference between recognition of poor care and necessary skills and capital to compensate for and change these interactions [16]. Therefore, even if the poor are able to recognize low quality of care or discrimination, their true disadvantage lies in their ability to develop and utilize cultural health capital. While several women describe in focus group discussions how they use social resources to assist in navigating the health system, most, however, have little or no cultural health capital because they lack past experiences with the healthcare system. The majority of women describe their lack of education and information to get to the facility, and that most of their information comes from their own social networks versus more formal or institutional sources. It is likely that their social network is also of low socioeconomic status, with similarly low levels of engagement or experience with formal healthcare [35]. Taken together, in addition to not having previous opportunities to acquire skills in the healthcare setting, women are also misinformed and may have lower expectations of care because of the social and cultural norms inculcated by friends and family.

Participants also described how providers set expectations and norms regarding behaviors in facilities. Patients with lower social standing may not always understand standard practices. Of importance, however, patients also blame themselves for their lack of knowledge rather than recognize that providers may improve communication to better inform patients of their choices and care processes. Such attitudes may create power disparities between providers and migrants that lead to abuse of patients [36]. In Ghana, a study of differential treatment between the “educated elite” and non-elite “villager” in healthcare settings hypothesizes that these differences are tied to larger social inclusion and exclusion of those who are educated versus non-educated in Ghanaian society at large [14]. Therefore, rather than blaming healthcare workers, it is more important to address underlying social determinants of health and understand where social disparities are produced and reproduced in larger institutions and society.

Existing social disparities and their reproduction in delivery care are also critical to consider with respect to migration. In the focus group discussions, women who report a lack of access to healthcare in their villages of origin again faced challenges in accessing care in Lucknow. Immigrants’ health in their destination community is shaped in part by their socio-economic status and other social characteristics in their villages of origin, as well as their opportunities in their destination [37]. Here, we find pre-migration disadvantage is reproduced. The majority of women in the sample come from rural areas. The rural-urban disadvantage is well-established in India, particularly related to maternity services such as antenatal care and delivery in a facility [20]. Because women lacked access to formal healthcare in their villages of origin, they were unable to develop cultural health capital, a needed resource for quality care in their destination.

A number of recommendations can be made from the data. First, future programs should increase opportunities for institutional engagement with marginalized urban residents. Several women reported in focus group discussions that they desired support from ASHAs in their community. Therefore, ASHAs could promote early antenatal care, familiarize them with available healthcare options, what to expect during the delivery experience, and how to navigate the healthcare system. Other women reported that they received information about reproductive and maternal health from ASHAs, suggesting that community health workers might be utilized to provide links to formal care for women with little or no prior experience with the public health sector. Lastly, this study found that well-positioned or connected community members in particular were helpful in increasing attention and prompt care. Patient advocates and continuous support during delivery, such as by a doula (woman trained to provide support to a woman during delivery), have been shown to improve patient satisfaction and decrease the likelihood of having intrapartum analgesia or an operative birth [38]. Therefore, specific training with community and family members to improve women’s cultural health capital should be developed.

There are a number of limitations to this study. First, this study represents an in-depth view of slums in one part of India. This study is focused in urban slum populations in Lucknow, Uttar Pradesh; therefore, it may not be representative of other parts of India outside slum populations, or even other slums in Uttar Pradesh. Future studies may include broader samples in order to understand whether expectations of care differ across a more diverse socioeconomic population of women. Second, this study was guided by cultural health capital theory; however, the survey is not able to measure levels of cultural health capital among participants because no validated measures have been developed. Future studies should develop quantitative measures of cultural health capital to understand the possible pathways that this may influence expectations and experiences with delivery care. Moreover, this study focuses on the experiences of women. However, it would be important to also include provider experiences in the future and to understand how physicians, nurses, and midwives are socialized to providing healthcare, reproducing societal inequalities within health facility settings, and the factors that may lead to mistreatment [39].

Despite these limitations, this study contributes to the larger literature on mistreatment among women in India by assessing prevalence of specific forms of mistreatment, and increasing understanding of how women understand and respond to mistreatment during delivery care. Given the renewed focus on women’s experiences during delivery [40], this study sheds light on potential strategies that women may use during care and highlights the importance of better understanding women’s expectation of care.

## Conclusions

This study demonstrates that poor women's true disadvantage lies in their lack of cultural health capital, including their lack of necessary skills to change negative interactions in health facilities. Providers set expectations and norms around behaviors during delivery, while women are often misinformed and may have low expectations of care. Future strategies should engage women, their families, and providers to promote women's cultural health capital to improve respectful care in facilities.

## Footnotes

1 This is the standard categorization in the Indian context for caste groups, with “other” encompassing the highest caste group, and the three others the lowest. While these may sound like stigmatizing terms, they hold meaning in this context as to the level of social and economic integration and potential discrimination that these groups face in society and therefore provide important information for understanding barriers to care.

## References

[1] Agarwal S, Sethi V, Srivastava K, Jha PK, Baqui AH (2010). Birth preparedness and complication readiness among slum women in Indore city, India. J Health Popul Nutr.

[2] Freedman LP, Kruk ME (2014). Disrespect and abuse of women in childbirth: challenging the global quality and accountability agendas. Lancet.

[3] Miller S, Lalonde A (2015). The global epidemic of abuse and disrespect during childbirth: History, evidence, interventions, and FIGO’s mother − baby friendly birthing facilities initiative. Int J Gynecol Obstet.

[4] Vogel J, Bohren M, Tunçalp Ӧ, Oladapo O, Gülmezoglu A. Promoting respect and preventing mistreatment during childbirth. BJOG Int J Obstet Gynaecol. 2015;n/a – n/a. 10.1111/1471-0528.13750 PMC5063112 26628382

[5] Kruk ME, Paczkowski M, Mbaruku G, de Pinho H, Galea S (2009). Women’s preferences for place of delivery in rural Tanzania: a population-based discrete choice experiment. Am J Public Health.

[6] Kruk ME, Kujawski S, Mbaruku G, Ramsey K, Moyo W, Freedman LP. Disrespectful and abusive treatment during facility delivery in Tanzania: a facility and community survey. Health Policy Plan. 2014;czu079. 10.1093/heapol/czu079 29304252

[7] Wagaarachchi PT, Fernando L (2002). Trends in maternal mortality and assessment of substandard care in a tertiary care hospital. Eur J Obstet Gynecol Reprod Biol.

[8] Kujawski S, Mbaruku G, Freedman LP, Ramsey K, Moyo W, Kruk ME (2015). Association between disrespect and abuse during childbirth and women’s confidence in health facilities in Tanzania. Matern Child Health J.

[9] Bohren MA, Vogel JP, Hunter EC, Lutsiv O, Makh SK, Souza JP (2015). The mistreatment of women during childbirth in health facilities globally: a mixed-methods systematic review. PLoS Med.

[10] Hulton LA, Matthews Z, Stones RW (2007). Applying a framework for assessing the quality of maternal health services in urban India. Soc Sci Med.

[11] Chaturvedi S, De Costa A, Raven J. Does the Janani Suraksha Yojana cash transfer programme to promote facility births in India ensure skilled birth attendance? A qualitative study of intrapartum care in Madhya Pradesh. Glob. Health Action [Internet]. 2015 [cited 2015 Dec 11];8. Available from: http://www.ncbi.nlm.nih.gov/pmc/articles/PMC4497976/. 10.3402/gha.v8.27427 PMC4497976 26160769

[12] Larson E, Hermosilla S, Kimweri A, Mbaruku GM, Kruk ME (2014). Determinants of perceived quality of obstetric care in rural Tanzania: a cross-sectional study. BMC Health Serv Res.

[13] Sen A (2002). Health: perception versus observation. BMJ.

[14] Andersen HM (2004). “Villagers”: differential treatment in a Ghanaian hospital. Soc Sci Med.

[15] Sitzia J, Wood N (1997). Patient satisfaction: a review of issues and concepts. Soc Sci Med.

[16] Shim JK (2010). Cultural health capital: a theoretical approach to understanding health care interactions and the dynamics of unequal treatment. J Health Soc Behav.

[17] Dubbin LA, Chang JS, Shim JK (2013). Cultural health capital and the interactional dynamics of patient-centered care. Soc Sci Med.

[18] Bourdieu P. The Forms of Capital. Handb. Theory Res. Sociol. Educ. [Internet]. New York: Greenwood Press; 1986. p. 241–58. Available from: http://eppl751su2012.wmwikis.net/file/view/Bourdieu.ch6.Forms.of.Capital.pdf/350871874/Bourdieu.ch6.Forms.of.Capital.pdf. Accessed 31 May 2016.

[19] Bourdieu P (1980). The Logic of Practice.

[20] International Institute for Population Sciences (IIPS) and Macro International. National Family Health Survey (NFHS-3), 2005–06: India: Volume I. Mumbai: IIPS; 2007.

[21] Madden EF (2015). Cultural Health Capital on the margins: Cultural resources for navigating healthcare in communities with limited access. Soc Sci Med.

[22] Balsa AI, McGuire TG (2003). Prejudice, clinical uncertainty and stereotyping as sources of health disparities. J Health Econ.

[23] Jewkes R, Abrahams N, Mvo Z (1998). Why do nurses abuse patients? Reflections from South African obstetric services. Soc Sci Med 1982.

[24] Department of Health and Family Welfare, Government of Uttar Pradesh. National Urban Health Mission [Internet]. Available from: http://nuhm.upnrhm.gov.in/urban/Lucknow/Slumlist.html. Accessed 31 May 2016.

[25] Srivastava NM, Awasthi S (2014). Breastfeeding practices for newborns among urban poor in Lucknow, northern India: A prospective follow-up study. Clin Epidemiol Glob Health.

[26] Bowser D, Hill K (2010). Exploring Evidence for Disrespect and Abuse in Facility-Based Childbirth: Report of a Landscape Analysis [Internet].

[27] Langer A, Campero L, Garcia C, Reynoso S (1998). Effects of psychosocial support during labour and childbirth on breastfeeding, medical interventions, and mothers’ wellbeing in a Mexican public hospital: a randomised clinical trial. BJOG Int J Obstet Gynaecol.

[28] Filmer D, Pritchett LH (2001). Estimating wealth effects without expenditure data—or tears: an application to educational enrollments in states of India*. Demography.

[29] Ulin PR, Robinson ET, Tolley EE. Qualitative Methods in Public Health: A Field Guide for Applied Research. San Francisco: John Wiley & Sons; 2004.

[30] Chalmers B, Omer-Hashi K (2002). What Somali women say about giving birth in Canada. J Reprod Infant Psychol.

[31] Small R, Yelland J, Lumley J, Brown S, Liamputtong P (2002). Immigrant women’s views about care during labor and birth: an Australian study of Vietnamese, Turkish, and Filipino women. Birth Berkeley Calif.

[32] Subramanian SV, Smith GD, Subramanyam M (2006). Indigenous health and socioeconomic status in India. PLoS Med.

[33] Mitra A (2008). The status of women among the scheduled tribes in India. J Socio-Econ.

[34] Gabrysch S, Lema C, Bedriñana E, Bautista MA, Malca R, Campbell OM (2009). Cultural adaptation of birthing services in rural Ayacucho, Peru. Bull World Health Organ.

[35] Cattell V (2001). Poor people, poor places, and poor health: the mediating role of social networks and social capital. Soc Sci Med.

[36] Akhaven S, Karlsen S (2013). Practitioner and client explanations for disparities in health care use between migrant and non-migrant groups in Sweden: a qualitative study. J Immigr Minor Health.

[37] Acevedo-Garcia D, Sanchez-Vaznaugh EV, Viruell-Fuentes EA, Almeida J (2012). Integrating social epidemiology into immigrant health research: A cross-national framework. Soc Sci Med.

[38] Hodnett E, Gates S, Hofmeyr G, Sakala C (2012). Continuous support for women during childbirth. Cochrane Database Syst Rev.

[39] D’ Oliveira AFPL, Diniz SG, Schraiber LB (2002). Violence against women in health-care institutions: an emerging problem. Lancet Lond Engl.

[40] WHO | WHO global strategy on people-centred and integrated health services [Internet]. World Health Organ. 2015 [cited 2015 Dec 22]. Available from: http://www.who.int/servicedeliverysafety/areas/people-centred-care/global-strategy/en/.

